# Characteristics and Risk Assessments of Mercury Pollution Levels at Domestic Garbage Collection Points Distributed within the Main Urban Areas of Changchun City

**DOI:** 10.3390/toxics9110309

**Published:** 2021-11-16

**Authors:** Zhaojun Wang, Yangjie Zhang, Lei Wang, Xu Li, Xuhang Zhou, Xiangyun Li, Mengping Yan, Qiuming Lu, Zhanhui Tang, Gang Zhang, Deli Wang

**Affiliations:** 1 School of Environment, Northeast Normal University, Changchun 130117, China; wangzj217@nenu.edu.cn (Z.W.); zhangyj415@nenu.edu.cn (Y.Z.); wangl788@nenu.edu.cn (L.W.); lix896@nenu.edu.cn (X.L.); zhouxh561@nenu.edu.cn (X.Z.); lixy869@nenu.edu.cn (X.L.); yanmp195@nenu.edu.cn (M.Y.); luqm834@nenu.edu.cn (Q.L.); tangzh789@nenu.edu.cn (Z.T.); wangd@nenu.edu.cn (D.W.); 2 State Environmental Protection Key Laboratory of Wetland Ecology and Vegetation Restoration, Changchun 130117, China; 3 Key Laboratory of Vegetation Ecology, Ministry of Education, Northeast Normal University, Changchun 130117, China; 4 Institute of Grassland Science, Northeast Normal University, Changchun 130022, China

**Keywords:** mercury, domestic waste, soil, children, risk indexes

## Abstract

The mercury that is released from the centralized treatment of municipal solid waste is an important source of atmospheric mercury. We chose the main urban area of Changchun as a representative area. Environmental factors such as total mercury content, temperature, wind speed, and other factors were measured in samples from the trash cans of two types of collection points (trash cans and garbage stations), the topsoil under the selected trash cans, and the ambient air above the selected trash cans. The potential ecological risks of mercury pollution were evaluated. The results showed that the mercury content levels of all sample types in the refuse transfer station were higher than the garbage cans and there were no significant differences observed between soil surface mercury and garbage cans. The mercury content levels in the atmosphere and the surface soil at the garbage collection points were found to increase along the cascade relationship of the garbage collection. However, there were no correlations observed between the atmospheric mercury content levels and the surface soil mercury content levels with the attachments and the sum of the former two. There were no correlations observed between surface soil and the attachments, or among the attachments, surface soil, and the atmospheric mercury content levels. The mercury content levels in the attachments, surface soil, and atmosphere of the garbage collection points in the study area were negatively correlated with the loop lines. Meanwhile, the potential ecological risk indexes of the garbage cans and garbage stations were found to be high. The chronic non-carcinogenic risks of mercury to children and adults were determined to be very low. The risks of mercury to children were higher when compared with adults. The highest non-carcinogenic risks of mercury pollution were determined to be within the central area of Changchun.

## 1. Introduction

Mercury is considered to be a global pollutant known to have strong toxicity, long-lasting and hidden pollution characteristics, easy migration abilities, and high bioaccumulation. After entering the atmosphere, mercury tends to circulate throughout the world before settling back to the Earth’s surface. It then may enter various ecosystems in order to complete various biogeochemical cycles [1,2,3,4].

Atmospheric mercury sources include both natural sources and anthropogenic sources. The research conducted by Mackay pointed out that the natural sources of atmospheric mercury include volcanoes, geothermal activities, soil, natural water, and plant transpiration, and the main form of mercury release is elemental Hg. The largest anthropogenic sources of atmospheric mercury are metal smelting and coal combustion, which potentially account for 45% and 38%, respectively, of the total mercury emissions. The known anthropogenic sources of atmospheric mercury include metal smelting, coal combustion, waste incineration, chlor-alkali industrial production, and various industrial products containing mercury [5,6,7,8].

The mercury released from municipal solid waste treatment centers is an important source of atmospheric mercury [9]. In 2015, the global total mercury emissions from waste incineration processes reached 14.74 tons. The output of municipal solid waste in China is high, and only 150 million tons of municipal solid waste are transported to landfills every year [4]. At the same time, China is also one of the largest mercury consuming countries in the world, with large amounts of mercury-containing waste products entering landfill sites [10]. Recent research results have shown that after 2012, the average mercury concentration levels in domestic waste in China’s provinces had reached 0.743 mg/kg, while the range of mercury content was between 0.080 and 2.560 mg/kg. In addition, according to the available statistical data, the total amount of mercury released in 2005 from landfill sites throughout the world reached 187 t [11]. Among those statistics, China accounted for 7.5%, reaching 14.1 t [12].

The mercury found in domestic waste mainly originates from various industrial products containing mercury. All types of mercury-containing waste products, including batteries, fluorescent tubes, mercury lamps, thermometers, and so on, are important factors causing mercury pollution [13]. The statistical results have shown that approximately 182 to 802 tons of mercury has been produced from batteries disposed of in domestic waste since 1992 [14]. At the present time, the biggest mercury consumption industry in China is the battery manufacturing industry [15,16]. Since 2017, with the implementation of the Minamata Convention on mercury, the relevant standards for mercury content in batteries have become stricter year-by-year, and mercury consumption in battery manufacturing industry has significantly decreased [15,17]. In addition, fluorescent lamp waste products are another important source of mercury pollution [18]. Recent research results have indicated that when a fluorescent lamp tube is broken, it will significantly increase the mercury content in many sections of a landfill, such as the garbage hoppers, exhaust funnels, and so on. Other sources of mercury pollution in landfills include medical devices and electronic and electrical equipment [17,19,20]. It is estimated that approximately 20% of medical equipment is transported to landfills every year. These waste products are mainly composed of sphygmomanometers and thermometers. Thermometers, along with some types of batteries, also have rather high mercury contents. Although the proportions of those substances in daily life are small, they make considerable contributions to mercury pollution. Additionally, due to their uneven distributions in domestic waste, the substances they contact may also become seriously polluted. It has been found that when compared with industrial products containing mercury, the mercury content levels of common domestic waste, such as kitchen and paper waste, are relatively low [21].

At present, there are three main treatment methods for dealing with domestic waste: landfills, incineration, and composting. Landfills have become the main means of domestic waste treatment in China due to their simple treatment methods and low costs [22].

**Figure 1:**
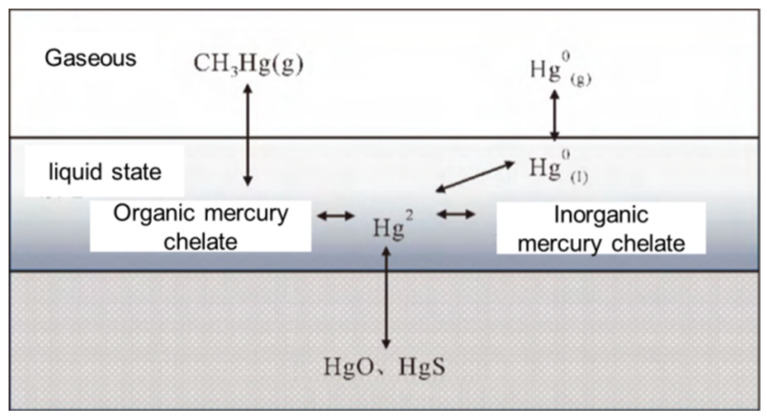
Mercury transformations in landfill sites.

The transformation processes of mercury in landfill sites are shown in Figure 1. The elemental mercury in garbage will volatilize into landfill gases after becoming broken down. In addition, the landfill gases will also contain some methyl mercury. In landfill sites, divalent mercury is generally inorganic mercury chelate. Mercury (Hg^2+^) in leachate reacts to form mercury sulfide (HgS) precipitation under anaerobic conditions and mercury oxide (HgO) precipitation under aerobic conditions. In addition, divalent mercury will become methylated and produce methyl mercury (CH_3_Hg). CH_3_Hg has strong biological toxicity and easily volatilizes, which will cause more extensive mercury pollution after entering the atmosphere. At the same time, mercury ions can also easily react with organic matter in waste products to form organic mercury chelate, which accounts for a high proportion of organic matter in waste, resulting in high content levels of organic mercury in landfill sites [23,24,25,26,27].

Municipal solid waste incineration processes have attracted the attention of the majority of economically developed cities. This is due to the advantages of their significant reduction effects, harmless nature, and high resource utilization, making incineration the preferred waste treatment method [28]. The main mercury pollution from MSW incineration is flue gas mercury, which has the following three main forms of Hg^0^, Hg^2+^, and HgP, with general proportions of 10 to 34%, 65 to 90%, and 1%, respectively [29,30]. Liu et al. [10] found that during the period ranging from 2010 to 2016, the input quantity of mercury from MSW incineration processes increased by 3.66 times, while the output of mercury also increased by 3.67 times.

Municipal solid waste (MSW) is closely related to the lifestyles of the residents, and the treatment of mercury in MSW has attracted wide attention. Mercury is known to have neurotoxicity and teratogenicity effects. Excessive amounts of mercury may lead to various health problems, such as corrosive bronchitis and interstitial pneumonia, and may damage the brain, lung, and kidney tissues of those exposed, as well as damaging central nervous system through the blood [31,32,33].

Therefore, it is necessary to evaluate the risks of mercury pollution at urban refuse collection points and formulate regional mercury environmental management and control measures [34]. Previous studies have focused on mercury emissions from municipal solid waste incineration plants. However, the characteristics of mercury emissions during the processes of municipal solid waste transportation are not clear and require further investigation. In the current study, the main urban area of Changchun City was taken as an example. The mercury emission concentration levels of the domestic waste collection points during the process of garbage collection and disposal in Changchun City were detected and analyzed in order to provide a basis for future environmental management and prevention measures for mercury release from urban domestic waste products. The study on the characteristics and generation of mercury in urban garbage collection points has very important theoretical and practical significance for accurately evaluating the health risks of exposed personnel, controlling urban mercury pollution, improving urban health and environmental quality, and preventing mercury pollution diseases.

## 2. Research Areas and Methods

### 2.1. Overview of the Study Area

The study area included the main urban area of Changchun City, situated between 43°05–45°15 N and 124°8–127°05 E. The study area was located in a north temperate zone of the middle latitude of the northern hemisphere. The Songliao Plains are located in the hinterland of the northeastern plains region of China [35].

#### 2.1.1. Natural Characteristics of the Study Area

Changchun City has the characteristics of a temperate continental monsoon climate. The annual average temperature is 4.6 °C. The highest temperature may reach 40 °C and the lowest temperature may reach −36.5 °C. The annual average precipitation ranges between 600 and 700 mm. The annual frost-free period is generally between 140 and 150 days, while the freezing period lasts approximately five months.

#### 2.1.2. Urban Characteristics

Changchun City has a total area of 20,565 km^2^, an urban area of 7557 km^2^, and a central built-up area of 506.33 km^2^. At the end of 2019, the total number of registered residents in the city was 7,538,000. The urban population reached 4,451,000, while that of the three counties (cities) (Nong’an County, Yushu City, and Dehui City) reached 3,087,000. Changchun’s gross domestic product is estimated at 590.41 billion yuan. The proportions of the three industrial structures are 5.9:42.3:51.8, respectively, while the contribution rates to economic growth are 4.1%, 79.8%, and 16.1%, respectively [36].

#### 2.1.3. Domestic Waste Generation

In 2015, the annual output of domestic waste in Changchun City (including the Shuangyang District) was 1.329 million tons. In 2019, the Chaoyang District generated approximately 776.1 tons of domestic waste per day, with the daily garbage output of the residents averaging 1.11 kg [37].

Changchun municipal solid waste (MSW) refers to the solid waste produced during the life spans of the Changchun residents. The main components include organic matter, coal ash, plastics, and so on. The MSW has the characteristics of large proportions, complex compositions, large variations, and low uniformity [38]. Previous research indicated that Changchun is a city within a coal-fired area and located in the north. The proportion of organic matter in its garbage is lower than in the cities within the gas-fired areas located in the south. In addition, the proportion of plastics is lower than observed in the south. However, the proportion of coal ash is higher than in southern cities [39].

Domestic waste products are required to go through three phases—collection, transportation, and treatment. Urban residents in Changchun City place domestic waste into garbage collection points, such as garbage cans. Garbage collectors collect bags of garbage and send them to nearby garbage transfer stations. Then, the garbage transfer stations collect and transport the waste to corresponding garbage treatment centers for treatment. At the present time, the treatment methods of garbage terminals include landfill sites, garbage collection, and garbage incineration, following the further classification and utilization of recyclable waste [40].

### 2.2. Research Method

#### 2.2.1. Layout of the Sample Plots and Sampling Points

**Figure 2:**
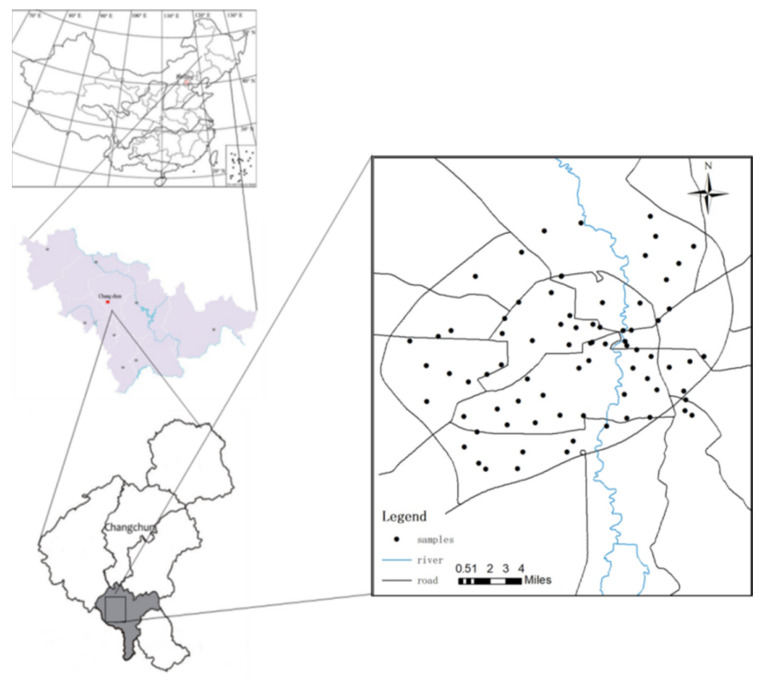
Layout of the sampling points.

In this study, the sampling areas in the main urban area of Changchun City were evenly distributed on a grid and then divided into 80 sampling units (2 km × 2 km). Then within each grid, the system evenly arranged the sampling points while considering the possibilities that during the actual sampling process some sampling points would be deleted or adjusted in a certain range. Finally, the location map of each of the sampling points was drawn according to the longitude and latitude of each sampling point, as detailed in Figure 2. A total of 78 sample points were obtained, including 54 barrel sample points, 22 station sample points, and 2 vehicle sample points.

#### 2.2.2. Sample Measurement Process

(1) Sample collection

On days with typical weather conditions, attachment buckets were taken to the sampling points in order to remove garbage, dead branches, fallen leaves, rocks, sand, and so on. The samples were placed into self-sealing bags and stored under dark conditions for standby purposes.

All of the samples were collected from the 0 to 10 cm topsoil layers under the refuse collection points of the sampling locations. All plants, dead branches, fallen leaves, rocks, sand, and other matter were removed. The samples were sealed and stored in polyethylene bags. The weights of each sample averaged approximately 500 g.

At each sampling point, the total mercury in the atmosphere was continuously measured for five minutes using a mercury meter and the average value obtained was considered to be the total mercury content in the atmosphere at each point. The GPS positioning and the surrounding environment of each sampling point were recorded. At the same time, any meteorological factors were measured, including the temperature, humidity, and wind speed. At the same time, the anthropic factors of corresponding garbage collection points were investigated and recorded.

In addition, the corresponding background values of the blank sample points in the urban clean green spaces were measured as references.

(2) Measurement method

In order to determine the total mercury in the atmosphere, a Russian Lumex RA-915 + Zeeman effect mercury analyzer was used for the real-time monitoring in the field. The data were obtained every 10 s, with an accuracy of 1 ng/m^3^.

In addition, in order to determine the total mercury in the soil, 50 to 200 mg of air-dried soil samples was measured using the single pass optical path sample pool of the Rumex ra-915 + mercury analyzer, combined with a UMA solid sample test unit. The air conveying speed was 4 L/min and the minimum detection limit of the solid samples determined by the instrument was 0.5 ng/g.

The quality control (QC) procedure was as follows: An XRF instrument was calibrated according to the NCRM (standard reference materials) of the People’s Republic of China (GBW07402a(GSS-2a)); the quality assurance (QA) process was implemented in order to ensure the accuracy of measurement results by repeatedly measuring at least one sample from each analysis group.

A temperature and humidity meter and an anemometer were used to synchronously measure the mercury content levels in order to identify the natural factors that may have influencing effects at each garbage collection point, and the corresponding data were recorded.

#### 2.2.3. Risk Assessment Method

(1) Potential ecological hazard assessment model: A potential ecological hazard index (*Er*) method [41] was used to assess the potential ecological hazard degrees of the attachments, surface soil, and atmospheric mercury at the garbage collection points. The calculation model was as follows:
  (1) $$Er=Tr\cdot C_{i}/C_{0}$$
  where *Er* represents the potential ecological risk coefficient of the mercury, *C_i_* is the measured value of mercury content, *C_0_* indicates the background value of the Hg, and *Tr* denotes the toxicity coefficient of the mercury (*Tr* = 40). The relationships between the potential ecological risk coefficients based on Er and the hazard degrees are shown in Table 1.
(2) Health risk assessment model: The RBCA (risk-based corrective action) calculation model proposed by the United States Environmental Program (EPA) was used in the present investigation of the health risk assessments of mercury in the attachments and topsoil of the study area [42]:
  (2) $$CDI=\frac{CS\times IR\times CF\times FI\times EF\times ED}{BW\times AT}$$

The meanings of each item are shown in Table 2.

Since mercury is a non-carcinogenic heavy metal [43], which can be calculated using a non-carcinogenic risk index (*HQ*) as follows:

*HQ* = *CDI*/*RfD* (3)

where *HQ* represents the possibility of non-carcinogenic risk, *CDI* is the chronic daily intake (mg/(kg·d)), *RfD* is the daily reference dosage (mg/(kg·d)), and the value of mercury is taken as 0.0001 mg/(kg·d) [44]. *HQs* can be utilized to assess the harm to human health during a person’s lifetime due to the intake of pollutants. It has been observed that under the same conditions, different people may be exposed to varying degrees of harm [45,46]. Therefore, this study chose to assess the health risks to adults and children, respectively.

When *HQ* > 1, there is considered to be a chronic non-carcinogenic risk. Meanwhile, when the *HQ* < 1, there is considered to be no risk. In this study, combined with the existing research materials, the exposure evaluation model parameters of the Changchun City garbage collection points were successfully determined, as detailed in Table 3.

#### 2.2.4. Data Statistics Method

In order to examine the distribution characteristics of the atmospheric mercury concentrations and the influencing factors of the garbage collection sites, SPSS 18.0 was used to analyze the sampling data, including the analysis of variance and correlation analysis. In the significance test, when *p* < 0.05, it was indicated that there was a statistically significant difference in the 95% confidence interval. Microsoft Excel 2016 software was adopted to process the data and draw the required tables. Origin Pro 2019b software was utilized to draw the statistical chart and ArcGIS 10.2 was used to draw the distribution map.

## 3. Result

### 3.1. Content Levels of Atmospheric Mercury at Refuse Collection Points in Different Functional Areas

#### 3.1.1. Mercury Content Levels in Garbage Containers (Stations, Bins)

In the present investigation, the average mercury content level in the attachments of garbage collection points in the main urban area of Changchun City was 32.68 ± 38.48 ng/g, ranging from 2.10 ng/g to 138.00 ng/g. The average mercury content level in the samples of the bucket attachments was 5.18 ± 3.12 ng/g, ranging from 2.10 ng/g to 12.00 ng/g. The average content level of mercury in the attachment samples was determined to be 47.34 ± 40.63 ng/g, with a concentration range of 4.70 to 138.00 ng/g. The average content of mercury in the blank sample was 1.93 ± 0.48 ng/g and the concentration range was 1.30 to 2.60 ng/g. Therefore, it was indicated from the results that the mercury content levels in the attachments of all the sampling points were higher than those of the blank sampling points, while the highest mercury content level was 71 times that of the blank sampling points. This study found that the mercury was enriched in the garbage containers in the main urban area of Changchun City. However, the mercury content values of the sampling points had fluctuated greatly, as shown in Table 4.

#### 3.1.2. Mercury Content Levels of the Surface Soil

The mean value of mercury in the surface soil layers of the garbage collection points in the main urban area of Changchun City was determined to be 33.15 ± 86.68 ng/g, ranging from 1.60 ng/g to 460.00 ng/g. The average content level of mercury in the samples was 18.57 ± 61.69 ng/g, ranging from 1.40 to 460.0 ng/g. The average content level of mercury in the attachment samples was 20.71 ± 30.01 ng/g, ranging from 1.60 to 159.00 ng/g. The average content of mercury in the blank sample was 1.93 ± 0.48 ng/g and the concentration range was 1.30 to 2.60 ng/g. The results obtained in this study revealed that the mercury content levels of 90% of the sample points were higher than those of the blank sample points, while the highest mercury content was 238 times that of the blank sample points, as shown in Table 5.

#### 3.1.3. Mercury Content Levels of the Ambient Air

The average atmospheric mercury content of the garbage collection points in the main urban area of Changchun City was confirmed to be 3.04 ± 2.79 ng / m^3^, with a range of 0.00 to 24.00 ng/m^3^. The average atmospheric mercury content of the bucket sampling points was 2.58 ± 1.83 ng/m^3^, with a concentration range of 0.00 to 17.00 ng/m^3^. The average content level of mercury in the air was determined to be 4.36 ± 4.19 ng/m^3^ and the concentration range was between 0.00 and 24.00 ng/m3. The average content level of atmospheric mercury in the garbage truck samples was 1.77 ± 0.43 ng/kg and the concentration range was 1.00 to 2.00 mg/kg. The levels of mercury in garbage truck samples were considered to be relatively low. The mean atmospheric mercury content of the blank sample was 2.12 ± 1.47 ng/m^3^ and the concentration range was between 0.00 and 5.00 ng/m^3^. Therefore, the results indicated that the mercury content levels of 62% of the sample points were higher than the blank sample points and the highest mercury content was eleven times the blank sample points, as shown in Table 6.

### 3.2. Environmental Factors and Health Management Strategies

#### 3.2.1. Environmental Factors

The average temperature of the barrel sampling points in the main urban area of Changchun City was determined to be 12.64 ± 4.47 °C and the temperature range was between 5 and 20 °C.

**Figure 3:**
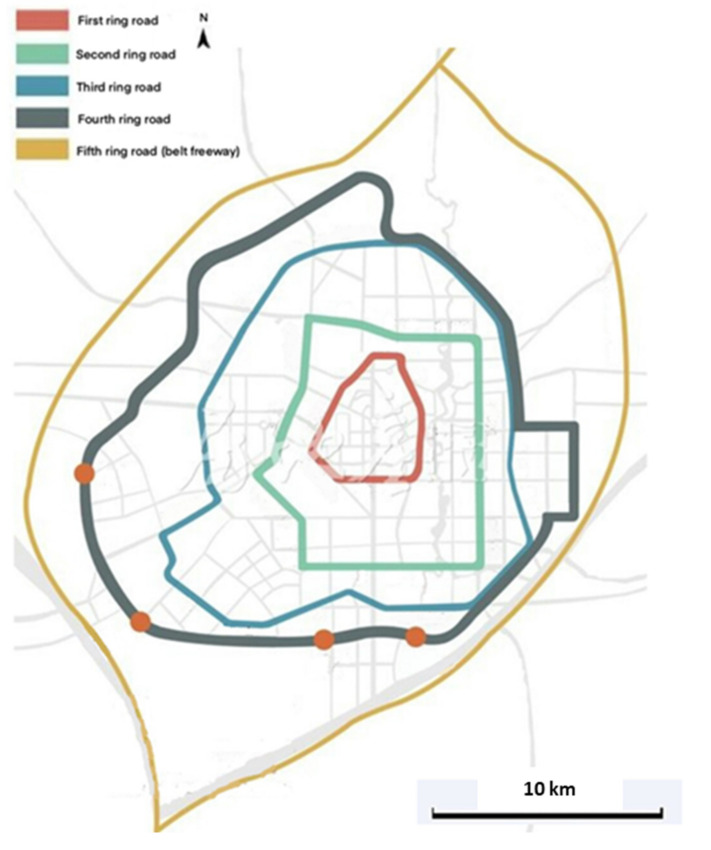
Ring road map of Changchun City.

The current urban form of Changchun City is a single-center structure. During the period ranging from 1992 to 2020, the built-up area of Changchun City was expanded by 518 km^2^. The shape of the urban area is relatively stable and is always circular, essentially expanding outward from a central circular design. The road network construction mainly includes ring lines and radial networks [49]. In accordance with the official media reports and the relevant research [50,51], the ring lines of the main urban area of Changchun City are detailed in Figure 3.

**Figure 4:**
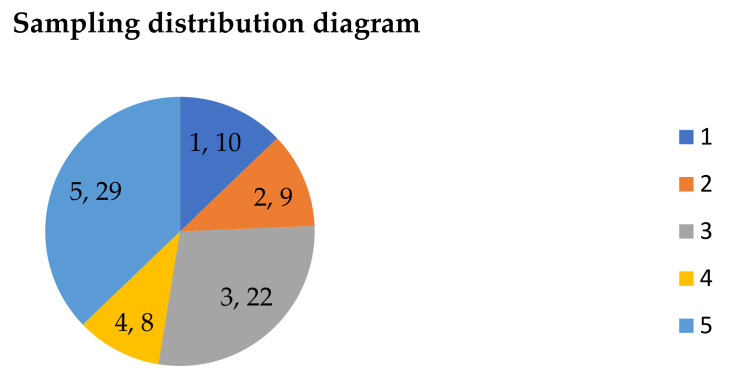
Sample points of each loop line.

According to the comparison results of the data shown in Figure 2 and Figure 3, there were ten sampling points in the first ring, nine in the second ring outside the first ring, 22 in the third ring outside the second ring, eight in the fourth ring outside the third ring, and 29 in the fifth ring outside the fourth ring, as shown in Figure 4 (in the figure, the front number represents the loop line and the rear number represents the number of sampling points).

#### 3.2.2. Health Management Strategy

Taking the Chaoyang District of Changchun City as an example, a management method involving bag collection, loading, unloading without landing, and closed transportation by compression truck was fully implemented in this study. There were 2040 front-line cleaning staff, 34 fully enclosed compression cars, 137 residential garbage sites, and 204 business sites [38].

**Figure 5:**
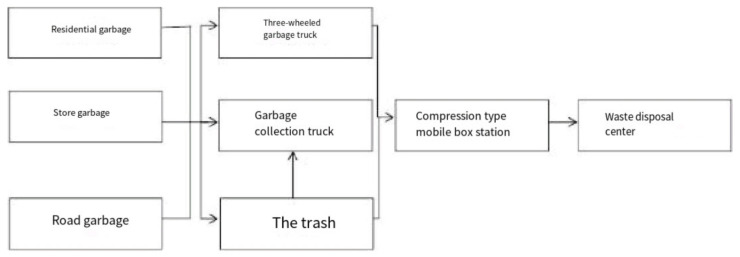
Flow chart of the garbage collection and transfer processes.

At the present time, there are mainly two methods of garbage collection and transportation in Changchun. The first involves garbage collection and transportation by buckets, which are collected and transported by multi-functional vehicles in the suburbs and shantytowns. In recent years, the old city of Changchun has been gradually transformed and the shantytown areas have been reduced. At the present time, the aforementioned type of collection and transportation method is only used in some areas of the urban–rural fringe. The second type involves the collection and transportation of garbage via compression vehicles. The cleaners collect the bagged garbage of residents and merchants at fixed points. The garbage is then then transported to the corresponding garbage transfer stations and loaded into compression vehicles. The compression vehicles continuously circulate the collection and transportation according to the corresponding times and routes of the areas and then transport it to garbage disposal sites [39,40], as shown in Figure 5.

The Changchun City sanitation workers are divided into groups, with each group having corresponding public areas and individual sharing areas. When working, each sanitation worker carries a long pole, garbage collection bag, and broom for the purpose of cleaning the personal sharing areas. When road garbage is discovered, the sanitation workers immediately sweep it into their garbage collection bags [52]. The garbage transfer station is cleared daily when the garbage output reaches 30 t. The station is cleared twice or even three times a day. In addition, the garbage transfer station is thoroughly cleaned twice during the summer months, and a simple cleaning is done during the winter.

### 3.3. Risk Assessments of Mercury Pollution Levels in Refuse Collection Sites

#### 3.3.1. Potential Ecological Risk Assessments

The total mercury data for different types of sampling points in different loop lines were utilized for this study’s potential ecological risk assessments. As can be seen in Table 3, Table 4 and Table 5, when the background value was used as the evaluation standard, the potential ecological risk degrees of the bucket samples ranged between medium and very strong, indicating strong potential ecological risks. The potential ecological risk indexes of the mercury pollution in the atmosphere, surface soil, and bucket attachments were found to be the highest in the first ring. Then, with the increases in the loop line, the potential ecological risk indexes of mercury pollution gradually decreased. It was observed that the farther the distance from the city center, the lower the potential ecological risk indexes of the mercury pollution. The potential ecological risk indexes of the atmospheric mercury pollution in the bucket sampling sites were weaker than those of the surface soil and attachments. In addition, the potential ecological risk degrees of the atmospheric mercury pollution in the sampling sites outside the first ring road were determined to be at a moderate level.

When the background value was used as the evaluation standard, the potential ecological risk indexes of the site ranged between slight and extremely strong, which also indicated strong potential ecological risks. The potential ecological risk indexes of the mercury pollution in the atmosphere and surface soil were determined to be the highest in the first ring and the lowest in the fourth ring. The potential ecological risks of mercury pollution of the surface soil in the second ring were found to be very strong, and the potential ecological risks of mercury pollution in the bucket samples obtained in the third ring were also very strong.

When the background value was used as the evaluation standard, the potential ecological risks of the vehicle samples were slight, indicating a relatively low potential ecological risk level, as shown in Table 7 and Table 8 (1–8 in the header indicates the sample type).

#### 3.3.2. Health Risk Assessments

The chronic non-carcinogenic risks of mercury to adults and children were also evaluated in this study. The data indicated that the non-carcinogenic risks of soil mercury were far less than 1 for both adults and children, which was within the maximum acceptable risk level recommended by the EPA (National Environmental Protection Agency). However, through comparison, it was found that the annual non-carcinogenic risk indexes of soil mercury for children were approximately ten times those for adults. The results showed that the non-carcinogenic risks to both adults and children of the surface soil mercury in the first ring of the bucket sampling site were higher than those outside the first ring. Furthermore, the non-carcinogenic risks to both adults and children of the surface soil mercury in the first ring of the station sampling site were higher than those outside the first ring. In summary, the non-carcinogenic risks of the surface soil mercury in the first ring and the third ring and the attachment mercury the first ring and the second ring were determined to be higher than those observed in the other sampling sites, as shown in Table 9.

## 4. Discussion

### 4.1. Distribution Characteristics of the Mercury Pollution in the Urban Refuse Collection Sites

#### 4.1.1. Mercury Content Levels at the Domestic Waste Collection Points

In this study, the investigation, sampling, experimental detection, and sample data results were analyzed using the ArcGIS 10.2 geostatistical analysis module, and a garbage collection point mercury content distribution map of the study area was drawn.

**Figure 6:**
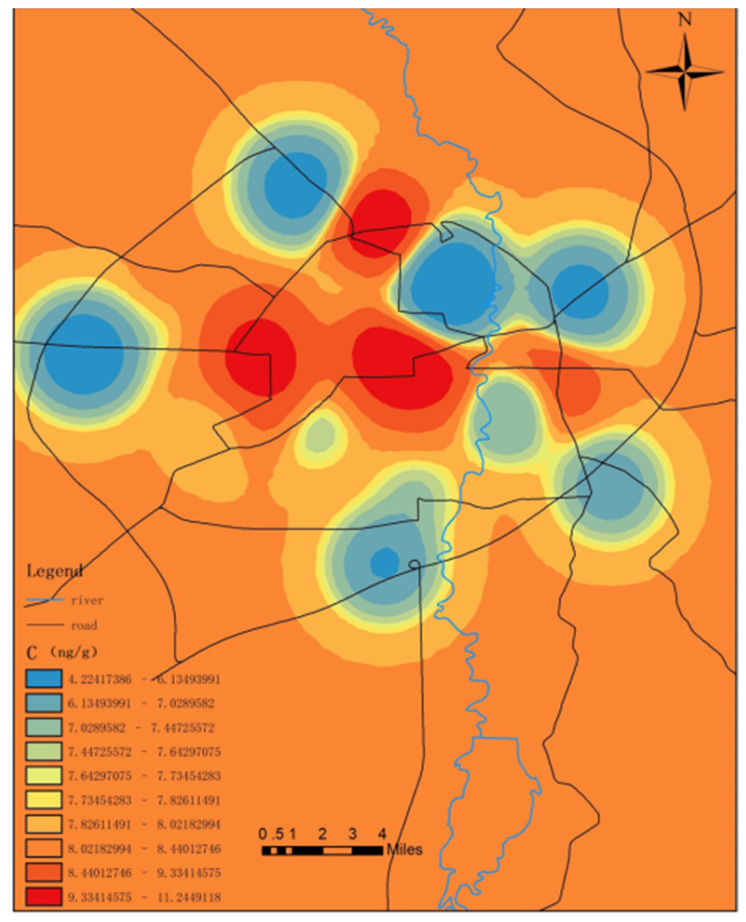
Distribution of mercury content in the attachments of the refuse collection points.

As can be seen in Figure 6, the spatial differences in mercury content were obvious. The patterns of the mercury content in the attachments resembled islands. It was found that the relatively high mercury content levels in the garbage collection sites were concentrated in the surrounding areas of medical institutions and traffic-intensive areas. However, the mercury content levels in wetland parks and urban suburbs were relatively low. The majority of the medical institutions were observed to be located in the center of the city and there was a lot of medical waste, including a large number of mercury thermometers and sphygmomanometers. During clinical diagnosis and treatment processes, mercury-containing thermometers and sphygmomanometers can be easily damaged, resulting in high levels of mercury pollution in medical garbage [53]. In contrast, the mercury content levels in Changchun City’s wetland parks and suburban areas were found to be relatively low.

**Figure 7:**
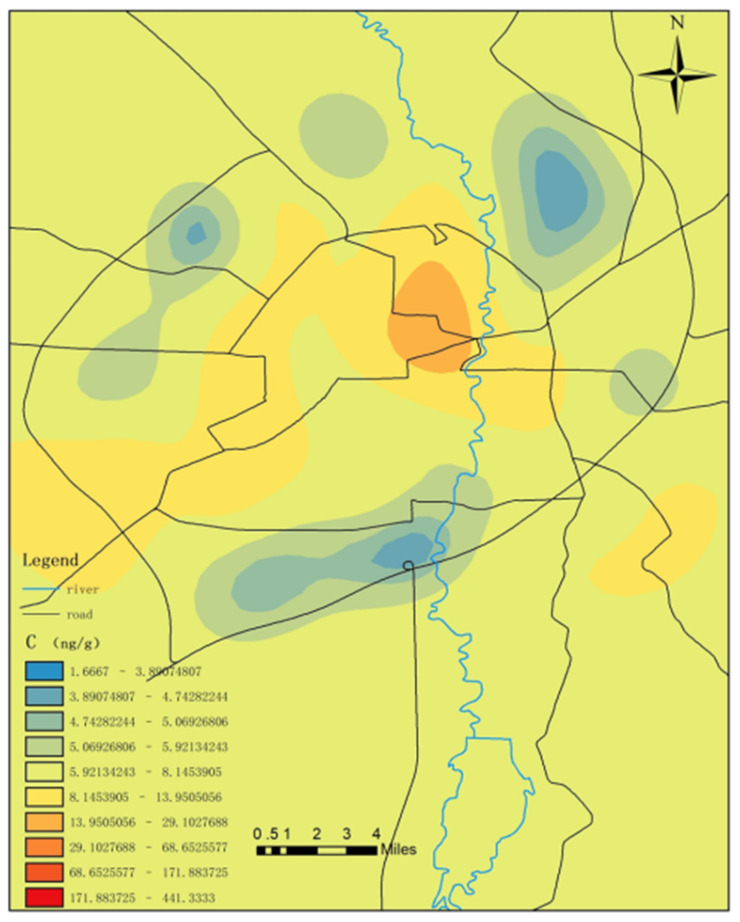
Distribution of surface mercury content at refuse collection points.

As detailed in Figure 7, the surface mercury content near the north of the central area (within the first ring road) of the study area was obviously high and the mercury content along the west ring road was also relatively high. In the central area, we observed a relatively large flow of people and vehicles. The output of garbage was high and the total amount of mercury-containing waste was also relatively high. As a result, the surface soil mercury content had the potential to also be relatively high in the central area of the city. Other relevant studies have shown that large- and medium-sized heavy industries (such as the No. 1 Thermal Power Plant), municipal smelters, and cement plants once operated in the central area. In recent years, the majority of those enterprises have moved out. However, the surface soil has accumulated large amounts of heavy metal mercury due to the previous long-term production activities [54].

**Figure 8:**
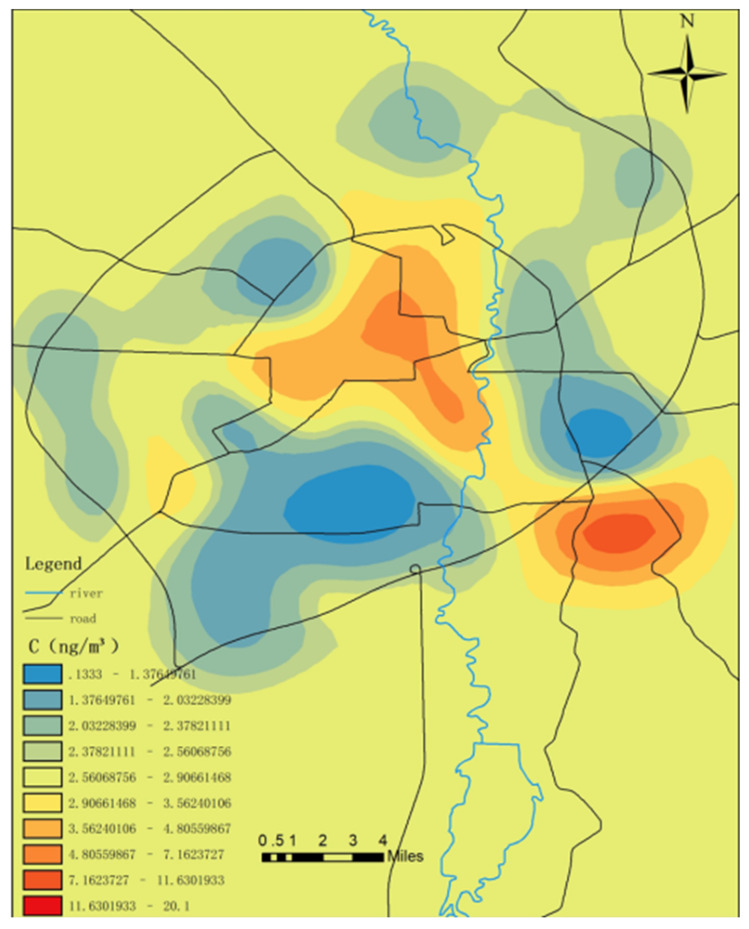
Mercury content levels in the atmosphere of the refuse collection points.

As detailed in Figure 8, the atmospheric mercury content levels in the central area of the Changchun garbage collection points and the Jingyue Development Zone were relatively high. The total amount of municipal solid waste was also relatively high, which may have been related to its relatively high mercury content. In 2010, Yang Zhongping and other researchers [53] determined that there were more than 64,000 streetlamps in Changchun, and nearly 6500 lamps were replaced annually [55]. The construction of the central urban area occurred earlier, and it was speculated that the long-term waste disposal of fluorescent lamps may also contribute to the mercury content in the central area. The two previously mentioned places are characterized by heavy traffic, vehicle congestion, concentrated exhaust emissions and slow diffusion, and relatively high mercury concentration levels [56].

#### 4.1.2. Comparison of the Mercury Content Levels between the Buckets and Stations

**Figure 9:**
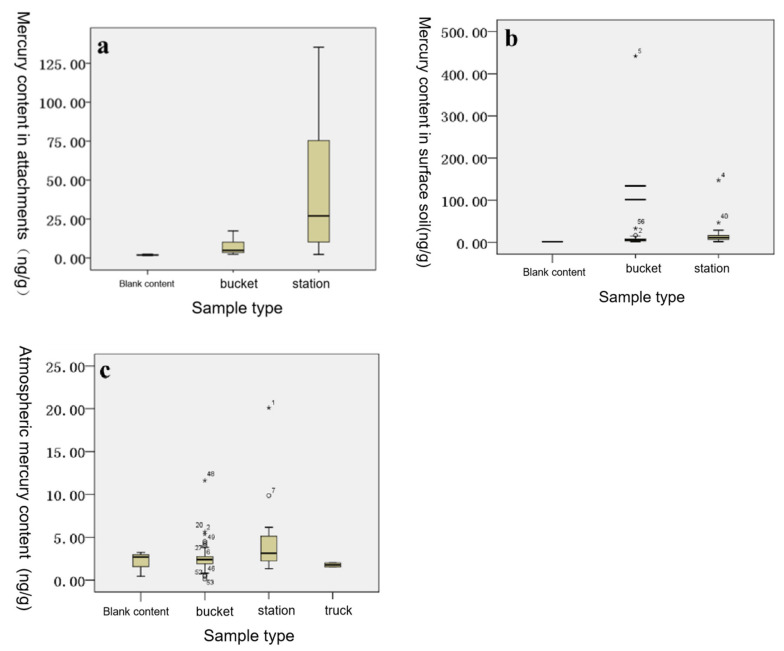
(**a**) Mercury content levels of the various types of attachments at different collection points. (**b**) Surface soil mercury content levels. (**c**) Atmospheric mercury content levels. “*” means extreme outlier.

As shown in Figure 9, the mercury content levels of the various samples obtained from the waste transfer stations were higher than those of the garbage cans. The mercury content levels of the attachments were 9.14 times those of the garbage cans and the differences were considered to be significant (*p* = 0.009). The atmospheric mercury content levels were 1.69 times those of the garbage cans and the differences were also considered to be significant (*p* = 0.010). There were no significant differences observed in the mercury content levels between the surfaces of refuse transfer stations and the garbage cans (*p* = 0.993). This may have been related to the regular cleaning and washing of the surfaces of the refuse transfer stations by the sanitation workers. In the studies conducted by Liu et al. [57], the results indicated that the atmospheric mercury concentration in the exhaust pipes of an anaerobic landfill unit in a landfill site was 10.09 ± 2.34 ng/m^3^ during the daytime (6:30 to 17:00). The atmospheric mercury concentrations of the landfill sites were determined to be higher than those of the refuse transfer stations during the daytime hours. Furthermore, the mercury content in the atmosphere of the garbage collection points increased along with the cascade relationship of the garbage collection, showing the order of landfills > garbage transfer stations > garbage cans. The research results obtained by international researchers were found to be higher than the atmospheric mercury content levels measured in this study. For example, Kim et al. [58] revealed that the atmospheric mercury emission concentration levels of waste incineration power plants in South Korea ranged from 1.96 to 4.71 μg/m^3^. In addition, Takahashi et al. [59] also determined that the atmospheric mercury emission concentration levels of waste incineration power plants in Japan ranged between 0.046 and 4.56 μg/m^3^.

Xie et al. [60] found that the mercury content level of the soil in a domestic waste incineration plant in northern China averaged 0.088 ± 0.064 mg/kg. Additionally, the mercury content levels of the surface soil around the landfill sites were higher than those measured in this study. As shown in Table 10, the mercury content levels of the soil around other waste incineration power plants both in China and internationally were higher than those measured in this study and displayed a concentration relationship of waste incineration power plant > waste transfer stations > garbage cans. The surface mercury content levels of the waste collection points were observed to increase along the cascade relationship of the waste collection, as shown in Table 10.

#### 4.1.3. Correlations between the Measured Mercury Content Levels of the Buckets, Surface Soil, and Atmosphere

This study completed a correlation analysis of the measured mercury content levels at the different sites in the study area. The results revealed that there were no correlations between the atmospheric mercury content and the surface soil, attachments, and the sums of those sites, which were all less than 0.2. In addition, there were no correlations observed between the surface soil and the attachments. In contrast, the results obtained in other relevant studies indicated that the atmospheric mercury concentrations were positively correlated with the mercury content in the soil, and the R value was 0.741 [66]. In the current investigation, the mercury content in the surface soil was observed to be less affected by the atmospheric mercury levels. Therefore, it was speculated that the mercury content in the surface soil was affected by external pollution sources, which may have potentially been the mercury-containing sewage from the garbage collection points.

### 4.2. Effects of Environmental Factors on Mercury Distributions in Refuse Collection Sites

**Figure 10:**
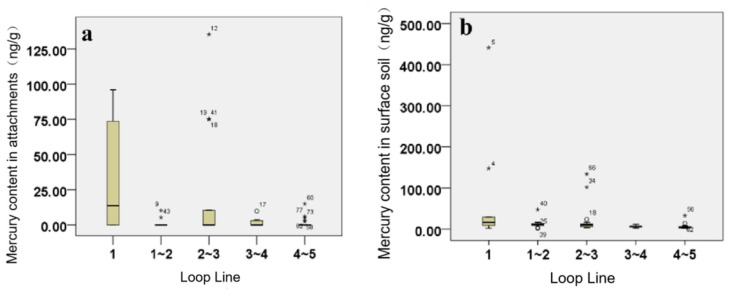

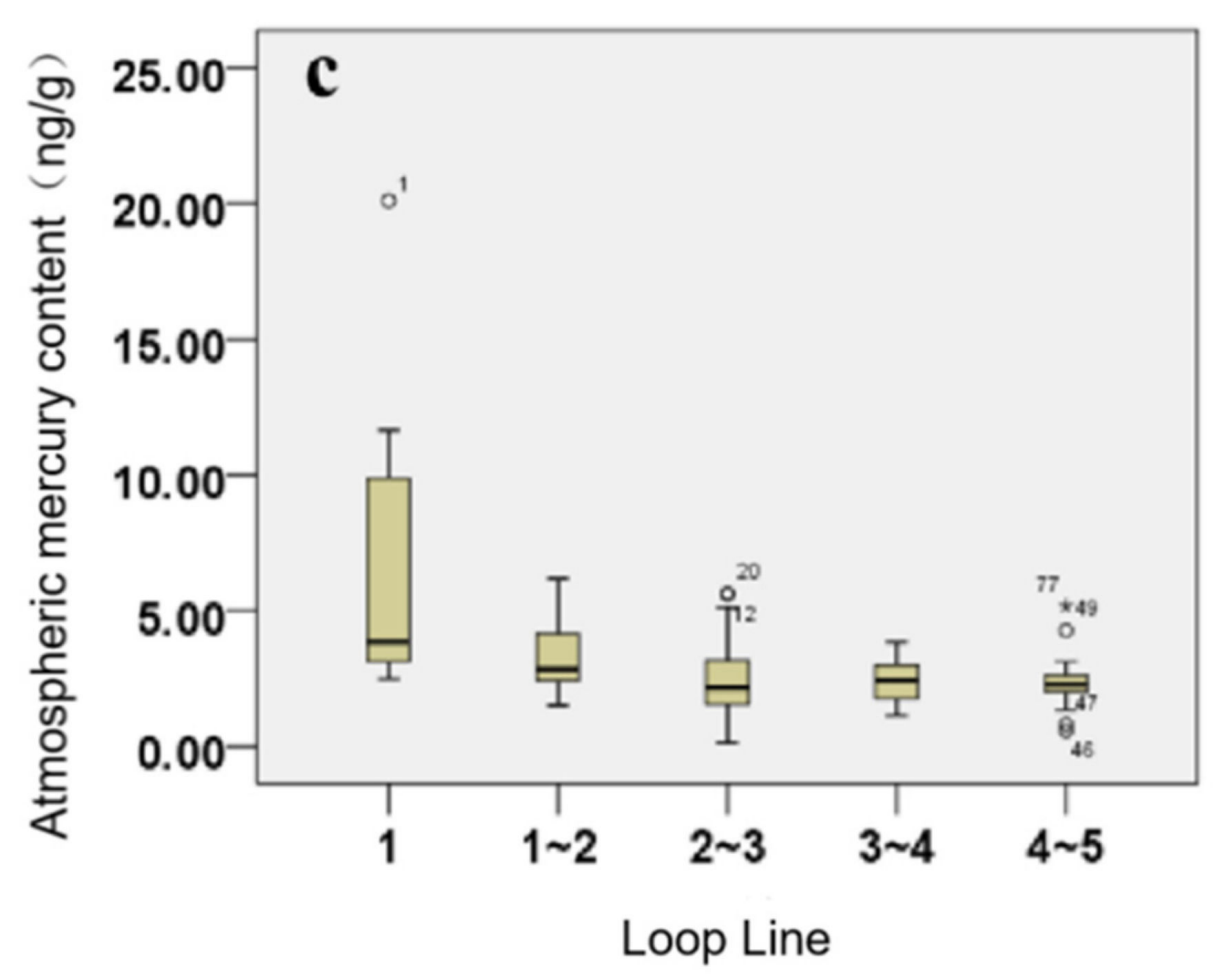
(**a**) Box chart of mercury content levels in the attachments of sample points within the different loop lines of Changchun City. (**b**) Mercury content levels of the surface soil sample points within the different loop lines of Changchun City. (**c**) Mercury content levels of the atmospheric sample points within the different loop lines of Changchun City. “*” means extreme outlier.

According to this study’s correlation analysis results, the mercury content levels in the attachments, surface soil, and atmosphere were negatively correlated with the loop lines (*R_1_* = −0.319; *R_2_* = −0.348; *R_3_* = −0.381). The mercury content levels decreased with the distance from the city center. A box chart was constructed in this study for the purpose of analyzing the mercury content levels in the attachments, surface soil, and atmosphere within the different loop areas. As shown in Figure 10, there were differences observed in the mercury content levels within the different loop areas for the attachments, surface soil, and atmosphere, and the highest mercury content was determined to be in the first (central) loop. The one-way ANOVA results revealed that the mean mercury content levels in the first ring, surface soil, and atmosphere were higher than those in other areas. However, no significant differences were observed, and there were no significant differences between the other examined areas. The mercury content levels in the attachments of the second ring outside the third ring were found to be higher than those of the third ring outside the fourth ring and the fifth ring outside the fourth ring. However, the differences were not considered to be significant.

This study found that the mercury content levels in the central part of Changchun City were significantly higher than those in the suburbs. The reasons may have been as follows. The mercury content in the urban garbage was partially historical due to the impacts of previous enterprises, and the construction of the central area had occurred earlier than that of the suburbs. Therefore, the garbage collection points had also been established earlier. Furthermore, the total amount of garbage in the central area was larger, resulting in higher levels mercury pollution. The industrial waste produced by urban construction activities in the central area may also have had certain impacts on the mercury content levels of the waste stations. However, the specific reasons for this will require further exploration.

### 4.3. Risk Assessments of Mercury Pollution in the Refuse Collection Sites

#### 4.3.1. Potential Ecological Risk Assessments

**Figure 11:**
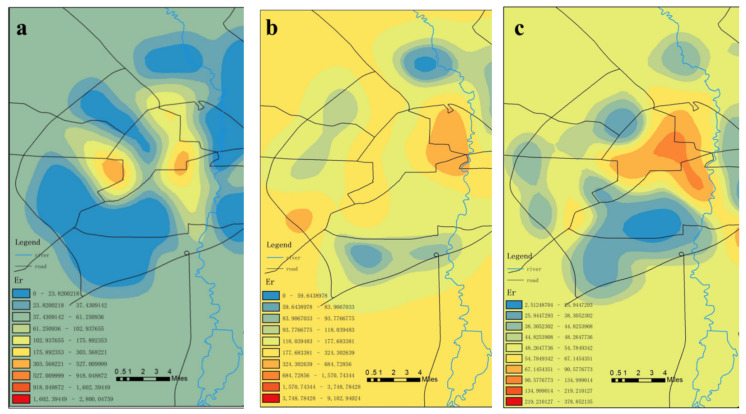
Potential ecological risk assessments: (**a**) distribution of the potential ecological risks of mercury pollution in the attachments; (**b**) surface soil; (**c**) atmosphere.

The data shown in Table 7 indicate that both the bucket samples and the station samples had potentially strong ecological risks with the background value as the evaluation standard. When the background value was used as the evaluation standard, the potential ecological risk index of the atmospheric mercury ranged from 2.51 to 219.27, the surface mercury index was determined to be 9102.92, and the mercury index ranged from 48.28 to 358.63. When the background value was used as the evaluation standard, the potential ecological risk index of the atmospheric mercury ranged from 25.13 to 378.85, the surface mercury index ranged from 37.81 to 3045.77, and the mercury index ranged from 46.90 to 2800.50. When the background value was used as the evaluation standard, the potential ecological risk index of atmospheric mercury ranged from 28.90 to 37.70. It can be seen in Figure 11 that there were strong potential ecological risks of mercury pollution in the attachments, soil surfaces, and atmosphere in the central area (within the first ring). There were also high potential ecological risk levels observed in the western section, as well as in the northern section of Changchun City. In addition to the high potential ecological risk levels in the central urban area, there were also high potential ecological risks observed in the Jingyue Development Zone.

In the present study, it was determined through the above analysis results that the potential ecological risk indexes of atmospheric mercury in the sample sites ranged from 2.51 to 378.85 when the background value was the evaluation standard. The surface soil mercury index ranged from 34.38 to 9102.92 and the mercury index of the bucket attachments ranged from 46.90 to 2800.50. Therefore, strong potential ecological risk levels were revealed in the study area. The mercury pollution in the study area was mainly concentrated in the central section. Therefore, increased attention should be paid to the mercury pollution control measures of the garbage collection points within that central location in Changchun City, as shown in Figure 11.

#### 4.3.2. Health Risk Assessments

**Figure 12:**
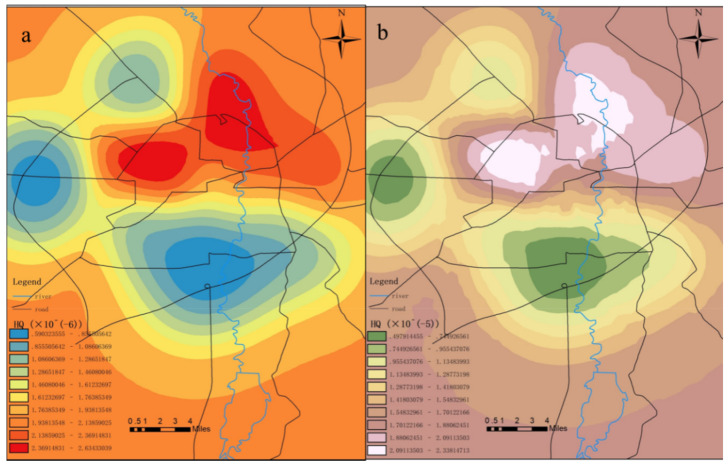
Distribution of the chronic non-carcinogenic indexes of mercury pollution for (**a**) adults and (**b**) children.

**Figure 13:**
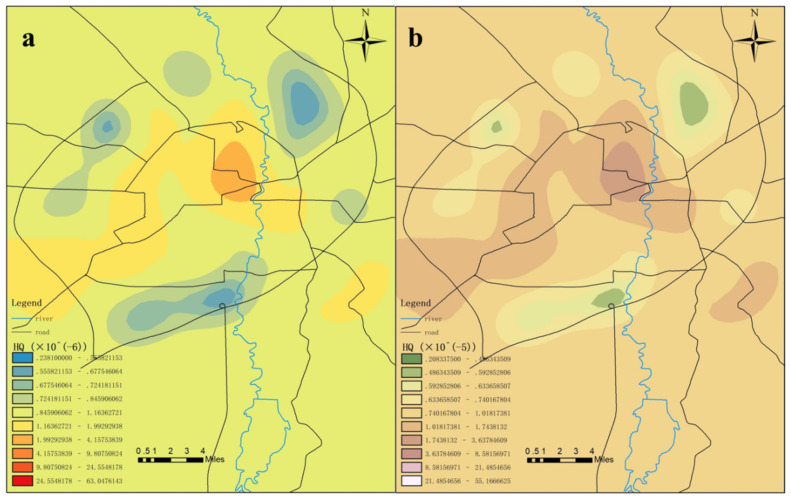
Distribution of the chronic non-carcinogenic indexes of mercury pollution in the surface soil for (**a**) adults and (**b**) children.

The data shown in Table 9 confirmed that the chronic non-carcinogenic risk indexes of mercury in the surface soil for adults ranged from 2.38 × 10^−7^ to 6.30 × 10^−5^. The chronic non-carcinogenic risk indexes for children ranged between 2.08 × 10^−6^ and 5.50 × 10^−4^. The chronic non-carcinogenic risk indexes of mercury for adults were between 1.44 × 10^−6^ and 2.48 × 10^−6^, and the risk indexes of chronic cancer in children were between 1.26 × 10^−5^ and 2.17 × 10^−5^. The chronic non-carcinogenic risks of surface soil mercury and mercury in the attachments for adults and children were found to not exceed the maximum acceptable level. However, due to the bioaccumulations of mercury, some cases showed that long-term exposure to low dosages of mercury would be harmful to human health [67]. Ninomiya et al. [68] found that 10 years after an experimental group were exposed to low dosages of mercury, such health problems as hypoesthesia, ataxia, and dysarthria were experienced. Therefore, the personnel working in the study area should also reduce their contact with relevant mercury-containing waste as much as possible. According to the comparison result for the data shown in Figure 12 and Figure 13, the chronic non-carcinogenic risks of soil mercury pollution to children were ten times that for adults. This was determined to be related to the physical fitness and living habits of the children to a certain extent. Children are still developing and their immune systems are generally weaker than for adults. In addition, special hand-to-mouth habits of children may cause them to more easily accidentally consume mercury from soil and garbage [69]. Therefore, it is important to pay close attention to the risks to children of mercury pollution. In addition, the maximum value of the chronic non-carcinogenic risk indexes of mercury in surface soil for adults and children appeared within the first ring, while the values of the risk indexes outside the fourth ring were lower. The maximum values of the chronic non-carcinogenic risk indexes of mercury in the surface soil for both adults and children appeared within the second ring and third ring, with the sample points within the first ring being relatively higher.

It was concluded based on the above analysis results that the measured content levels of mercury presented minimal chronic non-carcinogenic risks to children and adults, although vigilance will still be needed. It was evident that children were at higher risk than adults, mainly due to their higher accessibility to mercury in the soil, which could potentially be ingested into the body via mouth-to-mouth or food-to-mouth activities. Therefore, the exposure of children to mercury-containing waste should be reduced as much as possible, along with developing good health habits. In addition, since the mercury content levels in the surface soil and the mercury content levels in the attachments within the first ring displayed the highest non-carcinogenic risks to both adults and children, it was suggested that human contact at the garbage collection points in the first ring should be avoided as much as possible in order to reduce the non-carcinogenic risks of mercury contamination, as shown in Figure 12 and Figure 13.

## 5. Conclusions

In the present investigation, based on the analysis results and the discussion regarding the distribution characteristics and relationships of mercury content levels at the different types of garbage collection points in Changchun City, the impacts of relevant environmental factors on mercury distribution were analyzed. The results were as follows. The average mercury concentration level in the attachments of the refuse collection points was determined to be 32.68 ± 38.48 ng/g, with a range of 2.10 to 138.00 ng/g. The mean value of the mercury content in the surface soil was 33.15 ± 86.68 ng/g and the range was from 1.60 ng/g to 460.00 ng/g; The mean value of the atmospheric mercury was 3.04 ± 2.79 ng/m^3^, with a range of 0 to 24 ng/m^3^.

This study found that the mercury content levels of all types of samples at the refuse transfer stations were higher than those observed in the garbage cans, and there were no significant differences observed in the mercury content levels between the surface soil and garbage cans. The mercury content levels in the atmosphere and surface soil of the refuse collection points were found to increase along the cascade relationship of refuse collection, showing the order of landfills > refuse transfer stations > garbage cans. However, there were no correlations observed between mercury content levels of the atmosphere and the surface soil, attachments, and the sums of the former two. The R values were all less than 0.2. In addition, there were no correlations observed between surface soil and the attachments. There were also no correlations found among the attachment, surface soil, and atmospheric mercury levels. The results also indicated that the mercury content levels in the attachments, surface soil, and atmosphere of the garbage collection points in the study area were negatively correlated with the loop lines (*R_1_* = −0.319; *R_2_* = −0.348; *R_3_* = −0.381), and the mercury content levels in the attachments, surface soil, and atmosphere of the first ring were significantly higher than those in the other areas. The mercury content level in the attachments of the second ring outside the third ring was found to be higher than for the third ring outside the fourth ring and the fourth ring outside the fifth ring. The potential ecological risk indexes of the garbage cans and garbage stations were also high. There were strong potential ecological risks of mercury pollution identified in the central area (first ring) in the forms of surface mercury and atmospheric mercury pollution. There were also high potential ecological risks observed in the western and northern sections of Changchun City. In addition to the high potential ecological risks in the central area, there were also high potential ecological risks identified in the Jingyue Development Zone.

Therefore, based on this study’s findings, it was suggested that the following measures should be taken to reduce the potential ecological risks of mercury contamination:

1. The surfaces of garbage cans and garbage stations should be cleaned regularly and the internal attachments should also be cleaned in a timely manner;
2. The garbage collection points should be avoided as much as possible and the garbage containing mercury should be treated separately in order to avoid mercury entering the garbage treatment process.

The results of extensive research and analyses have shown that exposure to mercury presents minimal chronic non-carcinogenic risk to children and adults, although this still should not be ignored. It was found in this study that when compared with adults, the health risks of mercury contamination to children were much higher. Therefore, children should be monitored in order to avoid unhealthy exposure. The non-carcinogenic risks of mercury produced by the garbage collection points within the first ring of Changchun City were determined to be the highest. Therefore, it will be necessary to avoid contacting the surface soil and attachments within that region as much as possible.

## Figures and Tables

**Table 1:** Potential ecological risk analysis criteria.

Er	<40	40–80	80–160	160–320	>320
Potential ecological hazard degree	Trifling	Middling	Powerful	Strong	Extremely strong

**Table 2:** Parameter meanings and unit of exposure assessment model.

Exposure Evaluation Parameters	Meaning	Unit
*CS*	Soil mercury concentration	mg/kg
*IR*	Uptake rate	mg/d
*CF*	Conversion factor	10^−6^ kg/mg
*FI*	Uptake fraction	%
*EF*	Exposure frequency	d/year
*ED*	Exposure time	year
*BW*	Receptor weight	kg
*AT*	Average contact time	d

**Table 3:** Parameters of the exposure assessment model.

Exposure Evaluation Parameters	Adult Reference Value	Children’s Reference Value	Reference Value	Data Sources
*BW*/kg	70	16	—	EPA [47]
*IR*/(mg·d^−1^)	100	200	—	EPA [47]
*ED*/year	30	10	—	EPA [47]
*FI*/%	—	—	1.0	—
*EF*/d	—	—	365	EPA [47]
*CF*	—	—	10^−6^	EPA [48]
*AT*	—	—	Non-carcinogenic: *ED* × 365	EPA [47]

**Table 4:** Mercury content levels of the attachments in garbage containers (unit: ng/g).

Sample Type	Mean Value	Minimum	Maximum	Standard Deviation	Coefficient of Variation
Blank sample	1.93	1.30	2.60	0.48	0.28
Bucket	5.18	2.10	12.00	3.12	0.78
Station	47.34	4.70	138.00	40.63	1.77
Total	32.68	2.10	138.00	38.48	4.02

**Table 5:** Soil surface mercury content levels (unit: ng/g).

Sample Type	Mean Value	Minimum	Maximum	Standard Deviation	Coefficient of Variation
Blank sample	1.93	1.30	2.60	0.48	0.28
Bucket	18.57	1.40	460.00	61.69	11.68
Station	20.71	1.60	159.00	30.01	2.61
Total	33.15	1.60	460.00	86.68	8.14

**Table 6:** Mercury content levels of ambient air (unit: ng/m³).

Sample Type	Mean Value	Minimum	Maximum	Standard Deviation	Coefficient of Variation
Blank sample	2.12	not detected	5.00	1.47	0.74
Bucket	2.58	not detected	17.00	1.83	0.96
Station	4.36	not detected	24.00	4.19	1.47
Truck	1.77	1.00	2.00	0.43	0.24
Total	3.04	not detected	24.00	2.79	1.49

**Table 7:** Potential ecological risk indexes of the different sample categories.

Sample Type	Sample Type	1	1~2	2~3	3~4	4~5
Buckets	Atmosphere	116.23	52.78	40.17	48.38	42.05
	Surface soil	2431.98	149.88	434.91	125.70	122.62
	Attachments	227.93	—	214.49	141.04	72.18
Stations	Atmosphere	132.15	75.24	60.84	37.07	50.37
	Surface soil	807.28	451.12	294.15	76.32	87.09
	Attachments	1176.99	161.38	1537.96	46.90	217.94
Trucks	Atmosphere	—	—	—	28.90	37.70

**Table 8:** Potential ecological risk degrees of the different sample types.

Sample Type	Sample Type	1	1~2	2~3	3~4	4~5
Buckets	Atmosphere	Powerful	Moderate	Moderate	Moderate	Moderate
	Surface soil	Extremely strong	Powerful	Extremely strong	Powerful	Powerful
	Attachments	Strong	—	Strong	Powerful	Moderate
Stations	Atmosphere	Powerful	Moderate	Moderate	Weak	Moderate
	Surface soil	Extremely strong	Extremely strong	Strong	Moderate	Powerful
	Attachments	Extremely strong	Strong	Extremely strong	Moderate	Strong
Trucks	Atmosphere	—	—	—	Weak	Weak

**Table 9:** Annual non-carcinogenic risks of soil mercury for adults and children.

Sample Type	Sample Type	Evaluation Population	1	1~2	2~3	3~4	4~5
Buckets	Surface soil	a (×10^−6^)	16.84	1.04	3.01	0.87	0.85
		b (×10^−5^)	14.74	0.91	2.64	0.76	0.74
	Attachments	a (×10^−6^)	0.79	—	0.09	0.32	0.07
		b (×10^−5^)	0.69	—	0.08	0.28	0.07
Stations	Surface soil	a (×10^−7^)	5.59	3.12	2.04	1.06	0.72
		b (×10^−6^)	4.89	2.73	1.78	0.93	0.63
	Attachments	a (×10^−7^)	6.77	0.56	8.85	0.32	0.60
		b (×10^−6^)	5.93	0.49	7.74	0.28	0.53

Note: In the table, a represents the results of the adult evaluations and b represents the results of the child evaluations.

**Table 10:** Mercury content levels in domestic and international research cases (unit: mg/kg).

Region	North China [61]	Beijing [62]	Pearl River Delta of China [63]	Shenzhen Qingshuihe [64]	Italy Pisa [65]
Mercury concentration levels	0.14	0.088	0.081	0.058	0.17

## Data Availability

The data presented in this study are tested and processed by ourselves. The data can be published and used in journals.
